# *De novo *assembly of potential linear artificial chromosome constructs capped with expansive telomeric repeats

**DOI:** 10.1186/1746-4811-7-10

**Published:** 2011-04-15

**Authors:** Li Lin, Dal-Hoe Koo, Wenli Zhang, Joseph St Peter, Jiming Jiang

**Affiliations:** 1 Department of Horticulture, University of Wisconsin-Madison, Madison, WI 53706, USA

## Abstract

**Background:**

Artificial chromosomes (ACs) are a promising next-generation vector for genetic engineering. The most common methods for developing AC constructs are to clone and combine centromeric DNA and telomeric DNA fragments into a single large DNA construct. The AC constructs developed from such methods will contain very short telomeric DNA fragments because telomeric repeats can not be stably maintained in *Escherichia coli*.

**Results:**

We report a novel approach to assemble AC constructs that are capped with long telomeric DNA. We designed a plasmid vector that can be combined with a bacterial artificial chromosome (BAC) clone containing centromeric DNA sequences from a target plant species. The recombined clone can be used as the centromeric DNA backbone of the AC constructs. We also developed two plasmid vectors containing short arrays of plant telomeric DNA. These vectors can be used to generate expanded arrays of telomeric DNA up to several kilobases. The centromeric DNA backbone can be ligated with the telomeric DNA fragments to generate AC constructs consisting of a large centromeric DNA fragment capped with expansive telomeric DNA at both ends.

**Conclusions:**

We successfully developed a procedure that circumvents the problem of cloning and maintaining long arrays of telomeric DNA sequences that are not stable in *E. coli*. Our procedure allows development of AC constructs in different eukaryotic species that are capped with long and designed sizes of telomeric DNA fragments.

## Introduction

Artificial chromosomes (ACs) were first developed in budding yeast *Saccharomyces cerevisiae *through the cloning and assembling of three DNA elements: the centromere, telomeres and origins of replication [1]. The success of yeast artificial chromosomes (YACs) was a driving force for the development of artificial chromosomes in multicellular eukaryotes. Human artificial chromosomes (HACs) and plant artificial chromosomes (PACs) can not only provide important tools for studying chromosome structure and function, but also hold great potential as next generation vectors for human gene therapy and plant genetic engineering [2-4]. Development of both HACs and PACs have been reported after a decade long effort involving many laboratories [5-9].

Several different techniques have been developed to assemble AC constructs in mammalian and plant species. Most of these techniques have focused on combining centromeric DNA with telomeric DNA fragments. Origins of replication are poorly defined in higher eukaryotes but presumably exist throughout their genomes [10]. Thus, the centromeric and telomeric DNA used in AC constructs may contain sequence motives required for DNA replication. The most common approach for developing artificial chromosomes is using a cloned centromeric DNA fragment as the backbone of the constructs. YACs or bacterial artificial chromosomes (BACs) containing centromeric DNA were commonly used in construct development [2]. Subsequently, telomeric DNA fragments are added to the ends of the YAC or BAC insert [6,7,9]. This results in a DNA molecule containing a large centromeric DNA fragment capped with telomeric DNA from the targeted animal or plant species.

One of the main shortfalls in the current approaches of HAC and PAC assembly is the very short telomeric DNA fragments included in the constructs. Satellite repeats, including telomeric repeats, cannot be stably maintained in *E. coli*. Thus, if the HAC/PAC construct or part of the construct containing the telomeric DNA fragments is propagated in *E. coli*, any long arrays of the telomeric DNA may be partially or significantly deleted or rearranged. Due to this problem the lengths of the telomeric DNA of previously reported HAC/PAC constructs were all shorter than those of the native chromosomes, which can reduce the efficiency of artificial chromosome formation and affect the stability of resulting minichromosomes [11,12].

We sought a new strategy to circumvent this problem. Here we report the development of two telomeric DNA vectors that can be used to generate long telomeric DNA fragments up to several kilobases. We also developed a vector that can be combined with BAC clones containing large centromeric DNA inserts. The cloned centromeric DNA can be subsequently recombined with expansive telomeric DNA resulting in an *in vitro *system for the production of AC constructs. This AC assembly system allows the generation of AC constructs capped with telomeric DNA in different sizes. The technique can be applied in different plant as well as animal species.

## Results

### Development of a vector as a centromeric DNA backbone

**Figure 1:**
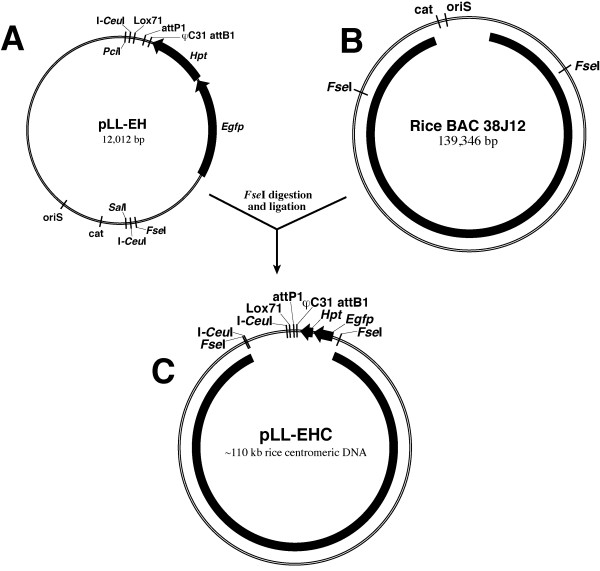
**Scheme for the development of the pLL-EHC vector containing a centromeric DNA fragment for use as the backbone in AC constructs**. (**A**) Structure of the pLL-EH vector. A 6,212-bp fragment between *Pci*I and *Sal*I was isolated from BAC vector pBeloBAC11. (**B**) Rice BAC clone 38J12 derived from the centromere of chromosome 8 [14]. (**C**) Ligation of *Fse*I-digested pLL-EH and BAC 38J12 resulting in the vector pLL-EHC.

We first developed the pLL-EH vector (Figure 1A). This vector (12,012 bp) consists of two DNA fragments. The first fragment (6,212-bp) was isolated from the BAC pBeloBAC11 [13] by double digestion with *Pci*I and *Sal*I (Figure 1A). This fragment contains all the genes required for stable propagation and maintenance of large DNA fragments in *E. coli*. The second fragment was synthesized containing a number of restriction sites for cloning and recombination. A hygromycin resistance gene (*Hpt*) and a reporter gene *Egfp *were also inserted into this fragment (Figure 1A). The attP1 site can be used for *in vitro *site-specific recombination with the attB1 site from telomeric DNA vectors. The lox71 and the φC31 attB1 sites can be used to insert additional DNA sequences into the vector or future potential artificial chromosomes of transgenic plants.

A BAC clone containing centromeric DNA can be ligated with pLL-EH to form a pLL-EHC vector (Figure 1C). Vector pLL-EHC, containing centromeric DNA, will represent the centromeric DNA backbone of the AC construct. We used a rice centromeric BAC 38J12 (Figure 1B) to develop our model pLL-EHC clone. BAC 38J12 contains an ~140-kb insert derived from the centromere of rice chromosome 8 (*Cen8*) [14]. The insert of this BAC spans the entire ~65-kb CentO centromeric satellite repeat array associated with rice *Cen8*. An 110-kb *Fse*I fragment of the insert, which spans the CentO repeat array, was released from 38J12 and ligated with a *Fse*I-digested pLL-EH vector to generate the pLL-EHC vector (Figure 1). This clone, now considered a centromeric seed clone, will subsequently be combined with telomeric DNA to form AC constructs.

### Development of two seed telomeric DNA vectors

It is well documented that long telomeric repeat arrays can not be stably maintained in *E. coli *[15]. Our strategy was to clone a short telomeric DNA fragment into seed telomeric DNA vectors. The seed vectors are used as templates to amplify long telomeric DNA fragments, which will then be ligated directly to the centromeric DNA backbone by *in vitro *site-specific recombination.

**Figure 2:**
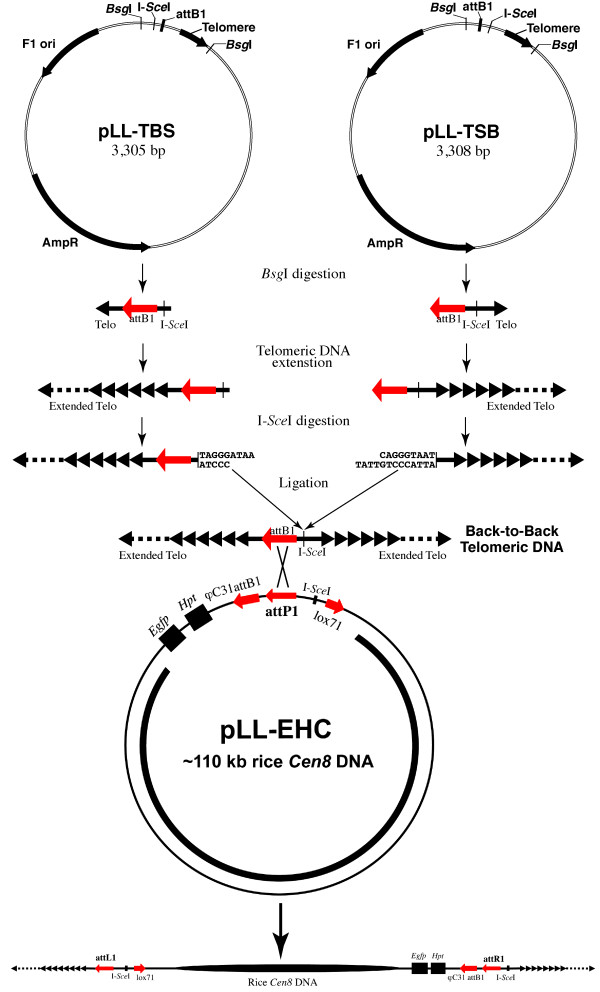
**Schematic diagram for generation of a linear AC construct**. Two seed telomeric DNA vectors pLL-TBS and pLL-TSB were used to generate expanded back-to-back telomeric DNA fragments. A subsequent *in vitro *recombination reaction between the pLL-EHC vector and the back-to-back telomeric DNA resulted in a linear AC construct.

A thermostable DNA polymerase from *Thermococcus litoralis *(Vent DNA polymerase) was used to generate long telomeric repeats from a short synthetic template/primer following a previously published protocol [16]. We cloned a 340-bp telomeric repeat fragment into the pGEM-T Easy vector. To develop a plasmid that contains a telomeric repeat segment flanked by the appropriate restriction and recombination sites required for future DNA manipulation, the telomeric DNA fragment originally cloned into pGEM-T Easy vector was subcloned into plasmid pTLT (see Materials and Methods). This resulted in two seed telomere vectors: pLL-TBS and pLL-TSB (Figure 2). Propagation of pLL-TBS and pLL-TSB in *E. coli *strain Top 10 resulted in partial deletions of the 340-bp telomeric DNA fragments. The stabilized pLL-TBS and pLL-TSB plasmids contained only ~120-bp of the telomeric repeats.

### Generation of long "back-to-back" telomeric DNA fragments

The two seed telomeric DNA vectors contain a homing endonuclease I-*Sce*I site, an attB1 site, and two *Bsg*I sites (Figure 2). The arrangement of the sites differs between the two vectors: *Bsg*I/I-*Sce*I/attB1/MCS/Telo/*Bsg*I for pLL-TBS, and *Bsg*I/attB1/I-*Sce*I/MCS/Telo/*Bsg*I for pLL-TSB (Figure 2). This arrangement causes the excised long telomeric DNA fragments derived from the two seed vectors to align in opposite orientation such that when later ligated to the ends of the centromeric DNA fragment, produces a linear molecule consisting of a central centromeric DNA element flanked by opposing telomere repeats (Figure 2).

**Figure 3:**
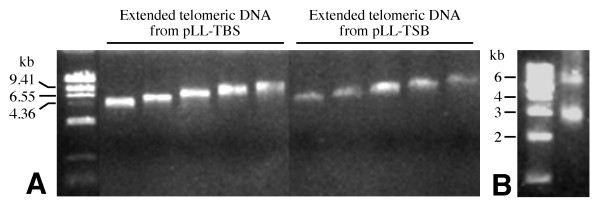
**Generation of back-to-back telomeric DNA fragments**. (**A**) Size-fractionated telomeric DNA fragments ranging in size from 4 to 10-kb derived from pLL-TBS and pLL-TSB, respectively. (**B**) An example of a 6-kb back-to-back telomeric DNA combined from two 3-kb telomeric DNA samples derived from pLL-TBS and pLL-TSB, respectively. The 3-kb band is the non-recombined telomeric DNA.

Digesting the pLL-TBS and pLL-TSB vectors with *Bsg*I released the short telomeric DNA inserts, including the attB1 site (Figure 2). The released DNA fragments were used as templates to generate long telomeric DNA fragments by unidirectional replication. This amplification step was accomplished by using Vent DNA polymerase that can catalyze short repeat expansion [16]. DNA fragments in the range of 2-10 kb were readily amplified using this approach (data not shown). The amplified telomeric DNA fragments were size-fractionated via gel excision to generate telomeric DNA varying in lengths (Figure 3A). The 5'-(TTTAGGG)_n_-3' and 3'-(TTTAGGG)_n_-5' DNA fragments of 2 to 5-kb in size were digested with I-*Sce*I and ligated to form back-to-back telomeric DNA (Figure 2, Figure 3B). Because the homing endonuclease I-*Sce*I recognizes asymmetric sites and the I-*Sce*I sites on the pLL-TBS and pLL-TSB seed vectors are arranged in an opposite orientation, a telomeric DNA fragment derived from pLL-TBS will only ligate with a fragment derived from pLL-TSB. Thus, the resultant back-to-back telomeric DNA will include two 2 to 5-kb synthetic telomeric DNA fragments in opposite orientation, one I-*Sce*I site, and one attB1 site (Figure 2).

### Development and characterization of linear AC constructs

The back-to-back telomeric DNA molecules were recombined with the pLL-EHC plasmid containing centromeric DNA to generate linear AC constructs. The recombination was accomplished through the attB1 site in the telomeric DNA molecule and the attP1 site in the pLL-EHC plasmid (Figure 2). This recombination resulted in a linear molecule consisting of the centromeric DNA fragment derived from pLL-EHC capped with expansive telomeric DNA at both ends. The attP1 and attB1 sites were converted into attL1 and attR1 sites after the recombination (Figure 2). The resulting linear molecules can be used directly for plant transformation with the *Hpt *gene used as the plant selection marker.

**Figure 4:**
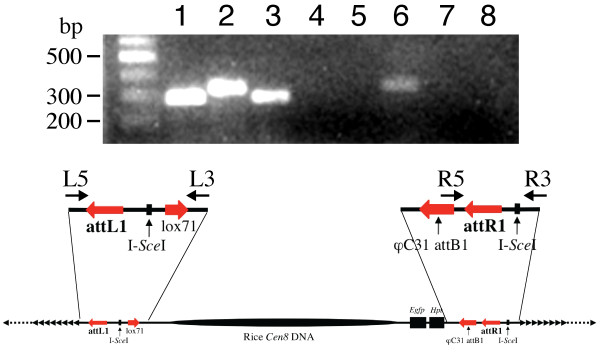
**PCR analysis of the linear AC constructs**. The primers specific to the left arm junction (L5 and L3) and right arm junction (R5 and R3) span the attL1 and attR1 sites, respectively. Lane 1: Left arm junction PCR using linear AC constructs capped with telomeric DNA at both ends. Lane 2: Right arm junction PCR using linear AC constructs capped with telomeric DNA at both ends. Lane 3: Left arm junction PCR using linear AC constructs capped with telomeric DNA at left arm only. Lane 4: Right arm junction PCR using linear AC constructs capped with telomeric DNA at left arm only. Lane 5: Left arm junction PCR using linear AC constructs capped with telomeric DNA at right arm only. Lane 6: Right arm junction PCR using linear AC constructs capped with telomeric DNA at right arm only. Lane 7: Left arm junction PCR using circular pLL-EHC DNA molecules. Lane 8: Right arm junction PCR using circular pLL-EHC DNA molecules.

**Figure 5:**
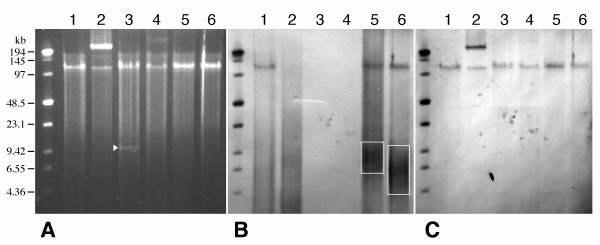
**DNA composition of the AC constructs**. (**A**) PFGE of AC constructs. (**B**) Southern blot hybridized with a telomeric DNA probe. (**C**) Southern blot hybridized with the rice centromeric satellite repeat CentO. Lane 1: Linear ACs capped with ~2 to 5-kb telomeric DNA at both ends. Lane 2: Mixture of pLL-EHC and back-to-back telomeric DNA without recombination. Note: the large bands associated with pLL-EHC did not hybridize to the telomeric DNA probe in (B). Lane 3: Linear molecules derived from recombination between pLL-EHC and linearized pLL-BKE (~10-kb of non-telomeric DNA, see Experimental Procedures). The arrowhead in (A) points to the non-recombined pLL-BKE molecules. Lane 4: pLL-EHC DNA after incubation with BP clonase. Lane 5: Linear AC constructs capped with ~5 to 10-kb telomeric DNA at the left arm only after recombination between pLL-EHC and expansive telomeric DNA from pLL-TBS. The red square in (**B**) includes the non-recombined telomeric DNA molecules. Lane 6: Linear AC constructs capped with ~5 to 10-kb telomeric DNA at the right arm only after recombination between pLL-EHC and expansive telomeric DNA from pLL-TSB. The red square in (**B**) includes the non-recombined telomeric DNA molecules.

To confirm the recombination between the back-to-back telomeric DNA fragment and the pLL-EHC plasmid specific PCR primers were designed from the junction regions based on the backbone sequences of plasmids pLL-EHC, pLL-TBS, and pLL-TSB (Figure 4). The linear AC constructs were isolated by pulsed field gel electrophoresis (PFGE). PCR amplification using the junction-specific primers resulted in DNA fragments matching expected sizes (Figure 4). The amplified PCR fragments were also confirmed by sequencing analysis (data not shown). We also developed linear constructs consisting of centromeric DNA capped with telomeric DNA at only one of the two ends. The single junction associated these constructs was also confirmed by PCR analysis (Figure 4). Southern blot hybridization analysis showed that only AC constructs with telomeric DNA attached at one or both ends hybridized to both telomeric and centromeric DNA probes (Figure 5).

### Cytological visualization of linear AC constructs

**Figure 6:**
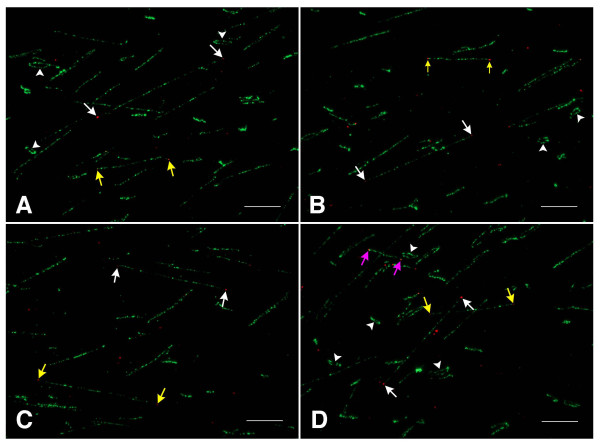
**Visualization of linear AC constructs by fiber-FISH using pLL-EHC (green) and telomeric DNA (red), as probes**. The two arrows of the same color in the four different images (**A**, **B**, **C**, and **D**) point to the two telomeric signals on the same AC constructs. Arrowheads point to some of the non-recombined circular pLL-EHC molecules. Note: the variability in size of the observed linear and circular DNA molecules is caused by non-uniform extension of the DNA molecules. Bars = 10 μm.

We used a DNA fiber-fluorescence in situ hybridization (fiber-FISH) technique to visualize the AC constructs resulting from the ligations between pLL-EHC and 4 to 8-kb back-to-back telomeric DNA fragments. The recombined DNA was directly spread on poly-lysine coated glass slides and hybridized with telomeric DNA probe (red) and pLL-EHC (green) probes. Linear DNA molecules hybridized with both probes were consistently detected using DNA samples from different ligation experiments (Figure 6). Non-recombined and circular pLL-EHC molecules were also observed. Some linear molecules showed no telomeric DNA signals or a signal at only one of the two ends. However, this is likely due to the resolution limitations of the fiber-FISH technique in which DNA sequences as short as few kilobases are often not detected as consistently as longer DNA fragments.

## Discussion

Methods for generating either *de novo *artificial chromosomes or engineered minichromosomes can be grouped into two broad categories: the "top-down" and "bottom-up" approaches, respectively. The "top-down" approach uses telomeric DNA to truncate a native chromosome thereby generating minichromosomes [17]. Selectable markers can be inserted into such minichromosomes to eventually be engineered into autonomous vectors highly similar to artificial chromosomes [18]. This "top-down" approach has been demonstrated in maize by truncating the supernumerary B chromosome [8]. While truncation of any normal chromosome in a diploid plant species may be lethal, the truncation of a normal chromosome can be achieved in a tetraploid genetic background of the targeted diploid species [8]. Nevertheless, it remains to be seen if such an approach can be applied to a broad set plant species. The "bottom-up" approach assembles AC constructs *in vitro *using cloned centromeric and telomeric DNA followed by transforming the target plant with the constructs [9].

The centromeric and telomeric DNA compositions of AC constructs can significantly affect the efficiency of artificial chromosome formation [2,19]. Linear centromeric DNA constructs capped with telomeric DNA generated HACs efficiently. However, a severe reduction in HAC formation coupled with an increase in integration events into human chromosomes was observed when the same constructs were not capped with telomeric DNA [11]. HACs that acquired long telomeres during *in vitro *propagation were more stable in mitosis than those with short telomeres [12]. These results indicate that adding long telomeric DNA to linear AC constructs has a major impact on the efficiency of artificial chromosome formation and the stability of the resulting minichromosomes. The telomeric DNA may protect the ends of the linear constructs in a similar function as the telomeres of normal chromosomes. Nevertheless, no specific study has been devoted to finding a relationship between telomeric end size in AC constructs and the efficiency of artificial chromosome formation.

The average telomere length of human fibroblasts varies from 5 to 10-kb [20]. However, BAC-based HAC constructs were capped by only 0.8 to 1.2-kb of telomeric DNA [19,21-23]. Similarly, only a 239-bp telomeric DNA fragment was included in the BAC-based PAC constructs in maize [9]. It was not specified in these previous reports as to why such short telomeric DNA fragments were included in HAC/PAC constructs. However, it is well known that satellite DNA, such as telomeric repeats [15], are generally not stable in bacterial plasmids, including BAC vectors [24]. Thus, even if BAC-based HAC/PAC constructs containing long telomeric DNA fragments can be developed, it remains unknown if such constructs can be maintained in *E. coli*. It is also interesting to note that putative de novo artificial chromosomes were recovered only 7 of the 450 transformation events using maize AC constructs capped with very short telomeric DNA [9].

It has been well documented that progressive shortening of telomeric DNA can lead to the loss of function of telomeres and the fusion of chromosomes [25]. Thus, it is reasonable to hypothesize that the amount of telomeric sequence capping AC constructs is important for the efficient formation of artificial chromosomes. We demonstrate that the instability of telomeric repeats can be circumvented by assembling of the AC constructs using expansive telomeric DNA, rather than cloned telomeric DNA. Since most plant species contain the same type of telomeric DNA [(TTTAGGG)_n_] [26], the pLL-TBS and pLL-TSB vectors can be used for telomeric DNA amplification and AC construct development in most plant species. In addition, telomeres normally terminate in a 3' single-strand G-rich overhang. This telomeric 3'-overhang is important for the formation of the T-loop, which is believed to protect chromosome ends from being recognized as broken DNA [27]. All previously reported AC constructs were not capped with telomeric DNA with this 3'-overhange structure due to means by which the telomeric DNA was cloned. Telomeric DNA fragments amplified from pLL-TBS and pLL-TSB do not undergo the cloning process and therefore can be modified to add this 3'-overhang structure before ligating with the centromeric DNA to produce linearized AC constructs.

Although AC constructs capped with sufficient length of telomeric DNA can be generated, PAC research will continue to face the major challenge of delivering such large constructs into plant cells. Currently, microprojectile bombardment is the most popular method to deliver such large constructs. Biolistic transformation of AC constructs will result in chromosomal integration and chromosome truncation events [9,28]. It will be interesting to investigate if application of AC constructs with expansive telomeric DNA will increase or decrase the frequency of such events.

## Materials and methods

### Development of backbone plasmids

The backbone of the pLL-EHC vector was constructed by linking two DNA fragments. The first fragment (6,212-bp) was isolated from the pBeloBAC11 vector [13] by digesting with *Sal*I and *Pci*I (Figure 1A). The second fragment was produced by multiple rounds of oligonucleotide-extension using six different primers. This fragment contains one I-*Sce*I site, two I-*Ceu*I sites, a lox71, an attP1 site, and a multiple cloning site (MCS) consisting of 16 unique restriction sites. The vector pLL-FF was developed by ligating the shared *Sal*I and *Pci*I sites between the synthetic fragment and the pBeloBAC11-derived fragment. A *Hin*dIII fragment containing the *Egfp *gene isolated from the Pk7GWIWG2D(II) vector (Invitrogen, Carlsbad, California) [29] was inserted into pLL-FF to create pLL-E. Finally, a *Bst*XI digested PCR fragment containing the *Hpt *gene, a plant selectable marker, from pHZWG7 [29], and a synthetic φC31 attP1 site were all inserted into PI-*Psp*I digested pLL-E to create pLL-EH (Figure 1). An ~110-kb fragment from the rice BAC OSJNBa0038J12 was then ligated into the *Fse*I site of pLL-EH to yield pLL-EHC.

To generate a telomeric DNA fragment, a PCR reaction was performed using a synthetic (TTTAGGG)_11 _DNA fragment as a template and a telomeric 25-mer (TTTAGGG)_3_TTTA) as a primer. The reaction was driven using Vent polymerase (1U) (New England Biolabs, Ipswich, Massachusetts) in a buffer containing 20 mM Tris-HCl (pH 8.8), 10 mM (NH_4_)_2_SO_4_, 10 mM KCl, 2 mM MgSO_4_, 0.1% Triton X-100, 1 mM dNTPs. The concentrations of both telomeric DNA fragments were 1 μM and the volume of the reaction was 5 μl. The amplified telomeric DNA fragments were cloned into the pGEM-T Easy vector (Promega, Madison, Wisconsin). One recombinant clone containing a 340-bp telomeric DNA fragment, pGEM-TT, was selected and confirmed by direct sequence analysis. The insert of the pGEM-TT plasmid was released using a *Nde*I and *Xba*I double digestion, blunt-ended using T4 polymerase (New England Biolabs), and subcloned into a *Pst*I-digested and blunt-ended pTLT plasmid. The pTLT plasmid is a modified pGEM-T Easy vector containing two additional *Bsg*I sites. This final clone was named pTLT-R11.

A PCR-based approach was used to insert the I-*Sce*I and attB1 sites into the pTLT-R11 plasmid. The following primers, which contain the attB1 and I-*Sce*I sites, were used in the PCR using pTLT-R11 as a template: TBS5' TTAGTCTCGAGACAAGTTTGTACAAAAAAGCAGGCTCTGCATGCCCTAAATCACTAGTGAATTCG; TBS3' TACTTCTCGAGACAAGTTTGTACAAAAAAGCAGGCTTGGTCTAGACCAAGATATCCTTGGC; TSB5' TTAGTCTCGAGTAGGGATAACAGGGTAATCTGCATGCCCTAAATCACTAGTGAATTCG. TSB3' TACTTCTCGAGTAGGGATAACAGGGTAATTGGTCTAGACCAAGATATCCTTGGC. The PCR fragments were digested with *Xho*I and self-ligated to yield the pLL-TBS and pLL-TSB plasmids.

### Synthesis of back-to-back telomeric DNA fragments

To generate long telomeric DNA fragments, the short telomeric DNA inserts were released from pLL-TBS and pLL-TSB plasmids by digesting with *Bsg*I. Unidirectional telomeric DNA extension was performed using a 5'-(tTTACCC)_12_-3' oligonucleotide. The oligonucleotides and the released plasmid inserts were mixed at a 1:2 ratio in a 100 μl PCR reaction containing 50 mM Tris-HCl pH 9.1, 16 mM NH_4_SO_4_, 3.5 mM MgCl_2_, 150 μg/ml bovine serum albumin (BSA), 250 μM dNTPs, Klentaq (5 U) (Clontech, Mountain View, California), and *Pfu *polymerase (0.03 U) (Stratagene, La Jolla, California). The extended DNA fragments were purified and treated with Mung bean nuclease at 30°C for 30 min to remove any single stranded DNA. The DNA fragments were then treated with calf intestinal alkaline phosphatase (New England Biolabs) at 37°C for 60 min to remove a phosphate group, ensuring that one DNA fragment from pLL-TBS and one from pLL-TSB would be ligated in a back-to-back direction.

The extended telomeric DNA fragments were separated on 0.7% low-melting agarose gel. Electrophoresis was performed over night at 37 V. DNA fragments of 2 to 10-kb were excised from the gels. The telomeric DNA was purified from the agarose and concentrated using a Microcon YM-50 spin column (Amicon, Houston, Texas) according to the manufacturer's instructions. Equal amounts of the size-fractionated telomeric DNA derived from pLL-TBS and pLL-TSB were mixed and digested with I-*Sce*I in a total volume of 200 μl for 3 h. The homing endonucleases were heat inactivated, and ATP (Epicentre, Madison, Wisconsin) was added to a final concentration of 1 mM. The telomeric DNA was ligated overnight at room temperature using T4 DNA ligase.

### Assembly of AC constructs

For the attB1 × attP1 recombination reaction, 500 ng of the attP1-containg pLL-EHC plasmid DNA and 100 ng of the attB-containing back-to-back telomeric DNA fragments were mixed with 4 μl each of 5 × BP clonase buffer and BP Clonase™ Enzyme Mix (Invitrogen), and adjusted to 20 μl with TE buffer. The mixture was allowed to react at 25°C for 16 h. After the recombinationreaction, the enzymes were inactivated by treatment with Proteinase K for 10 min at 37°C. Similar recombination reactions were also performed using the pLL-EHC vector and expansive telomeric DNA fragments derived from either pLL-TBS or pLL-TSB alone. This resulted in a polarized capping of the in linear centromeric DNA molecules with telomeric DNA at only one of the two ends.

The assembled linear AC constructs were separated by PFGE. The DNA band corresponding to the expected size of the linear AC constructs was excised from the gel and placed into 0.5 × TBE. The electro-eluted DNA was then dialyzed into ddH_2_O and concentrated using a Microcon YM-100 spin column (Amicon). The purified DNA fragments were used as a template for PCR analysis (see below). We developed a pLL-BKE plasmid as a control for artificial chromosome confirmation analysis. The pLL-BKE plasmid is a modified pLL-E vector with an attB1 site instead of attP1 site. Recombination between pLL-EHC and a linearized pLL-BKE (~10 kb), resulted in a linear molecule that is not capped with telomeric DNA.

Specific primers were designed from the junction regions between the pLL-EHC vector and the two telomeric DNA fragments as follow: L5 5'-TGATTTAGGGCATGCAGAGCCTGC-3', L3 5'-CTGTCAAGGGCAAGTATTGACATGT-3', R3 5'-TGATTTAGGGCATGCAGATTACCC-3', and R5 5'-TCATCTATGTTACTAGAGTACGCGC-3'. The positions of these primers are shown in Figure 6. PCR reaction mixtures contained 1 μM primers, 200 μM dNTPs, 0.01% gelatin, 2.5 mM MgCl_2_, 50 mM KCl, 10 mM Tris-HCl (pH 8.3), and 1 U rTaq (Takara Bio, Shiga, Japan) in a final volume of 20 μl. Following an initial denaturing step at 95°C for 5 min, 35 amplification cycles of 30 s at 95°C, 30 s at 60°C and 40 s at 72°C, were followed with a final incubation at 72°C for 7 min.

### Southern blot hybridization and fiber-FISH

Southern hybridization of DNA was performed using the digoxigenin (DIG) detection system (Boehringer Mannheim BV, Almere, The Netherlands). The probes used in the Southern hybridization were synthesized and DIG-labeled by random priming. The alkali-labile form of DIG-11-dUTP, the pRCS2 plasmid DNA insert containing the CentO repeats [30], and the *Not*I/*Nhe*I digested 140-bp telomere DNA fragments from pTLT-R11 were used as templates for random labeling with DIG. Hybridization conditions were carried out as recommended by the manufacturer.

Fiber-FISH analysis of assembled AC constructs was performed using published protocols [31]. An appropriate amount of target DNA resulting from ligations between pLL-EHC and 4 to 8-kb of back-to-back oriented telomeric DNA, was directly dropped on a poly-lysine coated glass slide and a 18 × 18 cover glass was carefully placed on the top of the DNA drop. Slides were hybridized with a telomeric DNA probe and the pLL-EHC plasmid. The signals were detected following the procedure of standard fiber-FISH [32].

## Competing interests

The authors declare that they have no competing interests.

## Authors' contributions

LL and JJ conceived and designed the experiments. LL, DHK, WZ, and JSP performed the experiments. LL and JJ analyzed the data and wrote the manuscript. All authors read and approved the final manuscript.
